# Enhancing Early Prediction of Gestational Diabetes Mellitus Through Data Augmentation and Feature Guidance: Model Development and Validation Study

**DOI:** 10.2196/85335

**Published:** 2026-05-25

**Authors:** Xiekun Chen, Zhifa Jiang, Dong Su, Xiaoping Chen, Aiping Chen, Zhen Zhang, Huabin Wang

**Affiliations:** 1 School of Computer Science and Engineering, Huizhou University, No. 46 Yanda Avenue, Huizhou, Guangdong, 516007, China, 86 18217267715; 2 Cyberspace Institute of Advanced Technology, Guangzhou University, Guangzhou, Guangdong, China; 3 Obstetrics and Gynaecology, Huizhou First Maternal and Child Health Care Hospital, Huizhou, Guangdong, China; 4 School of Mathematics and Statistics, Huizhou University, Huizhou, Guangdong, China

**Keywords:** gestational diabetes mellitus, GDM, data augmentation, feature enhancement, machine learning, SHAP analysis

## Abstract

**Background:**

Early prediction of gestational diabetes mellitus (GDM) is critical for improving maternal health outcomes. However, predictive models are often challenged by limited early-pregnancy samples, severe class imbalance in datasets, and complex interrelationships among clinical features.

**Objective:**

This study aimed to develop and evaluate a unified dual-dimensional enhancement framework integrating data augmentation and feature engineering. By addressing data imbalance and leveraging medical prior knowledge, this framework significantly improves early GDM prediction performance.

**Methods:**

We proposed a framework combining Generative Adversarial Network (GAN)–based data augmentation with large language model–inspired feature engineering. GAN sampling was used to generate clinically plausible synthetic minority class samples to mitigate data imbalance. The large language model was guided to organize features into domains (eg, basic demographics, metabolic syndrome, and core liver biomarkers) and generate higher-order composite features, integrating medical prior knowledge. Machine learning models were subsequently developed, and interpretability analyses were performed using Shapley additive explanations to identify key predictors.

**Results:**

This study used a final analytical cohort of 8214 pregnant women, divided into dataset A comprising 966 out of 5251 (18.4%) participants with GDM, and dataset B comprising 598 out of 2963 (20.2%) participants with GDM. The random forest model enhanced by Tabular Variational Autoencoder–based feature augmentation demonstrated the best performance. On the test dataset, it achieved a recall of 0.7559, an accuracy of 0.8444, and an area under the receiver operating characteristic curve (AUROC) of 0.8873. Statistical evaluation confirmed that the Tabular Variational Autoencoder method significantly outperformed the baseline (Cohen *d*=2.894; *P*<.001) and the Conditional Tabular Generative Adversarial Network method (Cohen *d*=1.637; *P*=.02) in recall enhancement. Shapley additive explanations analysis identified the following 5 features as the most influential predictors: fasting blood glucose, the composite feature (fasting blood glucose+triglycerides)×prepregnancy BMI, activated partial thromboplastin time, leukocyte count, and neutrophil count.

**Conclusions:**

The proposed dual-dimensional enhancement framework effectively alleviates data limitations and captures complex feature interactions in early GDM prediction. This strategy not only improves model performance, particularly in recall, but also provides interpretable biological evidence to support rapid clinical screening, stratified management, and early intervention in pregnancy.

## Introduction

Gestational diabetes mellitus (GDM), defined as glucose intolerance first detected during pregnancy, has a persistently high incidence and typically occurs in the second or third trimester, leading to adverse maternal and fetal outcomes [1]. Women with GDM face increased risks of gestational hypertension, infections, postpartum hemorrhage, preterm birth, and later progression to type 2 diabetes mellitus [2]. However, efficient identification and management remain challenging due to predictive uncertainty [3]. In China, the prevalence of GDM has been reported to reach 24.24%, emphasizing the critical clinical importance of early and precise prediction to enable timely and effective intervention [4]. Although the Oral Glucose Tolerance Test (OGTT) at 24‐28 weeks is the standard diagnostic method, maternal hyperglycemia may already harm the fetus before this stage, and mid-to-late pregnancy interventions often miss the optimal window to prevent irreversible risks [5-7]. Evidence suggests that early assessment between 12 and 14 weeks, and no later than 20 weeks, is optimal, and that lifestyle interventions in early pregnancy can reduce GDM risk [8].

The increasing maturity of machine learning (ML) has promoted its use in early risk prediction and risk stratification in GDM-related clinical scenarios. For example, our previous study applied ML models to predict postpartum dyslipidemia among women with GDM, illustrating the potential value of ML-based approaches in this population [9]. ML models can enable precise screening of high-risk populations and provide in-depth analyses of early-pregnancy risk factors to support timely interventions. Since 2010, related research has grown rapidly, with developed predictive models emphasizing different aspects [10-12]. Wu et al [13] used ML algorithms to develop a GDM risk stratification model for Chinese pregnant women, assessing GDM risk before 16 weeks’ gestation. Cooray et al [14] used multivariable regression to integrate multiple indicators and constructed the PeRSonal (Prediction for Risk-Stratified Care for Women with GDM) model, achieving favorable results. Belsti et al [15] compared several ML algorithms, including logistic regression (logit model), k-nearest neighbors, and Gaussian Naïve Bayes, to develop GDM risk prediction models and identified the optimal model for GDM prediction. Zhu et al [16], in a multicenter longitudinal cohort study, validated that steroid hormone indicators used in early- and midtrimester Down syndrome screening can be used to construct GDM prediction models [16]. Lyu et al [17] applied logistic regression to select predictive factors; the final model incorporated 9 clinical and biochemical features and demonstrated good predictive performance in the training cohort.

The early prediction of GDM faces 3 major challenges. First, the scarcity of early-pregnancy data is a core bottleneck limiting model performance, as primary health care institutions often lack complete prenatal testing protocols before 14 weeks’ gestation, thereby restricting the acquisition of sufficient high-quality clinical data [18]. Second, severe class imbalance, manifested as a disproportionately low proportion of positive cases, undermines model recall and markedly increases the risk of missed diagnoses among high-risk pregnant women [19]. Third, complex interrelationships exist among clinical indicators across glucose-lipid metabolism, coagulation, and other physiological systems. These complex interactions limit the effectiveness of traditional expert-driven feature engineering, ultimately weakening model generalizability. Collectively, these challenges impede the development of robust, clinically relevant, and generalizable early prediction models for GDM.

To address the aforementioned challenges, this study proposes a unified dual-dimensional enhancement framework, with specific strategies encompassing the following approaches:

1. We introduce a Generative Adversarial Network (GAN)–based data augmentation method to address class imbalance in GDM prediction. Unlike conventional oversampling or undersampling approaches that often sacrifice either accuracy or recall, the proposed method substantially improves recall while maintaining high accuracy.
2. Inspired by large language models (LLMs), we propose a feature enhancement framework that deeply integrates medical prior knowledge with data distribution patterns, thereby further improving predictive performance.
3. We use interpretable analyses grounded in information theory and game theory to investigate model outputs, elucidate and evaluate the decision process of the GDM model, and validate the effectiveness of the feature enhancement framework. This work links model-derived findings with clinical mechanisms underlying GDM.

Our unified framework bridges the semantic gap between purely data-driven algorithms and medical prior knowledge. Traditionally, feature engineering in early GDM prediction relies heavily on highly subjective, manual expert selection. Instead, our study innovatively uses LLMs as knowledge distillers. By feeding raw statistical data distributions into the LLMs, we establish a novel paradigm that translates unstructured pathological knowledge from medical literature into structured, systems biology–guided feature aggregations. Furthermore, the synergy between GAN-based data augmentation and LLM-inspired composite features provides a dual-dimensional enhancement.

Our experimental results demonstrate that the model established using the dual-dimensional enhancement framework, particularly the random forest model enhanced by Tabular Variational Autoencoder–based feature augmentation (TFRFM), achieved the best performance in predicting GDM. The model attained a recall of 0.7559, an accuracy of 0.8444, and an area under the receiver operating characteristic curve (AUROC) of 0.8873, indicating that TFRFM substantially enhances recall without compromising accuracy. This effectively reduces the risk of missed diagnoses among high-risk pregnant women and significantly outperforms conventional sampling methods.

**Figure 1.**
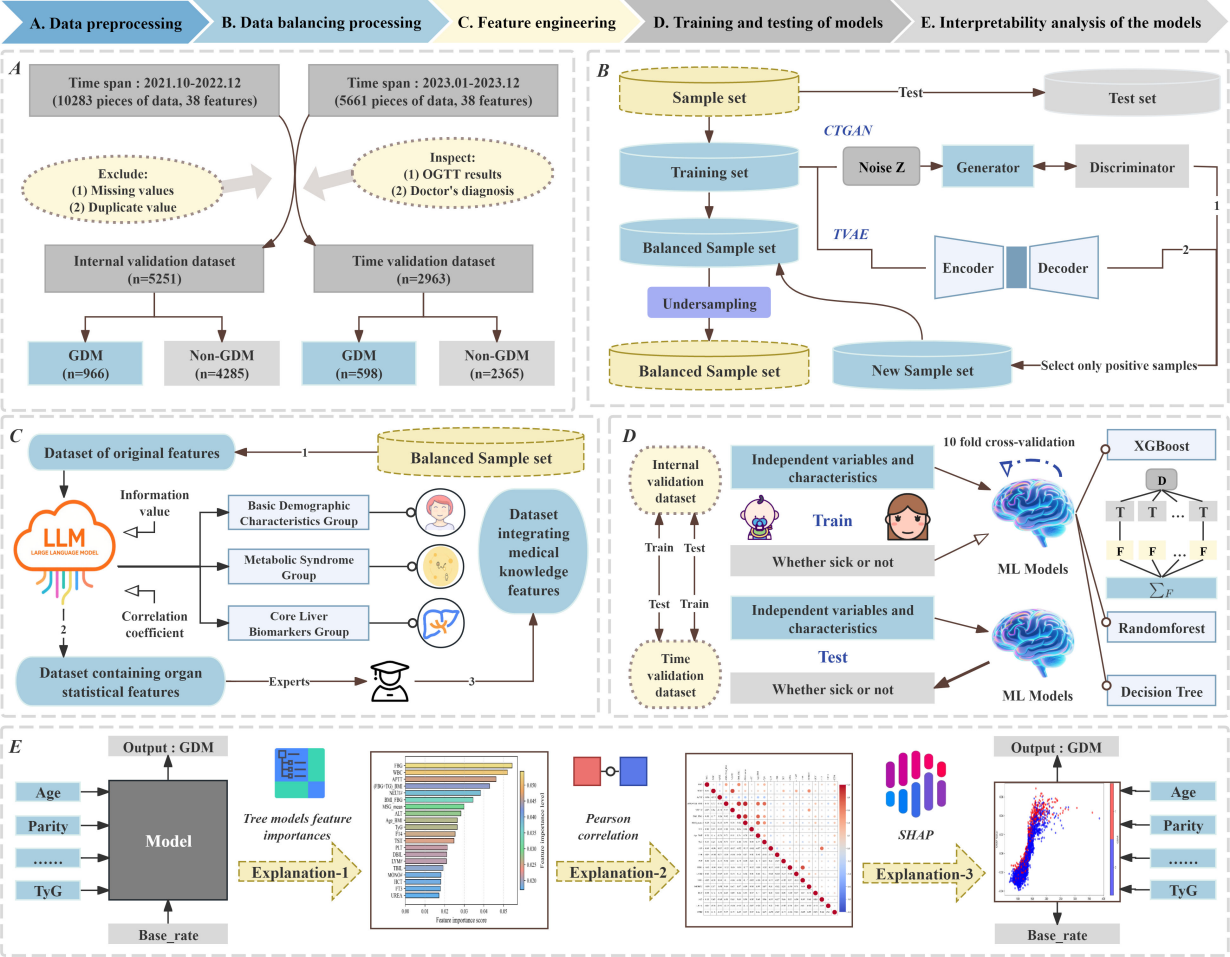
Research framework: (A) data preprocessing, (B) data balancing processing, (C) feature engineering, (D) training and testing of models, and (E) interpretability analysis of the models. CTGAN: Conditional Tabular Generative Adversarial Network; GDM: gestational diabetes mellitus; LLM: large language model; ML: machine learning; OGTT: Oral Glucose Tolerance Test; SHAP: Shapley additive explanations; TVAE: Tabular Variational Autoencoder; TyG: triglycerides glucose index; XGBoost: Extreme Gradient Boosting.

The research workflow is illustrated in Figure 1. The remainder of this paper is organized as follows: the “Methods” section describes the data sources, data preprocessing methods, and the construction and validation of the ML models. The “Results” section presents the evaluation of the ML models and the results of interpretability analyses. “Discussion” section discusses the clinical significance of early-pregnancy laboratory indicators for GDM prediction and the impact of improved model recall on GDM management. Finally, the “Conclusion” section concludes the study.

## Methods

### Data Source and Preprocessing

This study enrolled 15,944 singleton pregnant women who delivered at Huizhou First Maternal and Child Health Hospital from October 2021 to December 2023 (10,283 cases in dataset A and 5661 cases in dataset B). These women were aged ≥18 years, underwent blood tests in the first trimester (<14 wk of gestation) and the second trimester (24‐28 wk of gestation), and completed a 75-g oral glucose tolerance test at 24‐28 weeks to identify whether they had GDM. Women with a history of diabetes, impaired fasting glucose, prepregnancy lipid-lowering medication use, or miscarriage or induction of labor before 28 weeks of gestation were excluded. The diagnosis of GDM was based on the criteria of the International Association of Diabetes and Pregnancy Study Groups (IADPSG) Consensus Panel, which defines GDM as meeting any of the following: fasting blood glucose (FBG) ≥5.1 mmol/L, 1-hour postload blood glucose ≥10.0 mmol/L, or 2-hour postload blood glucose ≥8.5 mmol/L [20].

The study variables include maternal demographic characteristics such as maternal age, prepregnancy BMI, parity, and gestational weight gain; blood test results during pregnancy and the puerperium, including total cholesterol, triglycerides (TG), OGTT results at 24‐28 weeks of gestation, blood routine, and coagulation function test results.

The preprocessing was conducted in three steps: (1) integration and cleaning, which involved removing irrelevant indicators and invalid OGTT records; (2) deletion of abnormal zeros and missing values; and (3) deduplication of rows and columns, with row-level deduplication based on IDs and column-level removal of redundant features using Pearson correlation coefficients.

### Data Augmentation Strategy Based on GAN Sampling

Clinical data on GDM exhibit a marked class imbalance. Let the original datasets be denoted as $D_{A}$ and $D_{B}$, and define the imbalance ratio ρ as the ratio of the number of minority class samples to that of majority class samples. In this study, $\rho_{A}$ and $\rho_{B}$ were 0.23 and 0.25, respectively, indicating a severely imbalanced dataset. Directly using the original datasets for modeling would lead to insufficient learning of minority class samples, typically manifested as a low recall rate.

To address this issue, 5 data balancing strategies were compared [21]: no resampling (baseline), undersampling, oversampling, Conditional Tabular Generative Adversarial Network (CTGAN), and Tabular Variational Autoencoder (TVAE). An optimized GAN-based minority sample generation approach was proposed to ensure both distributional consistency and clinical plausibility of the generated samples.

### Traditional Sampling

In handling imbalanced datasets, traditional sampling methods primarily alleviate the impact of class imbalance on model performance by adjusting the number of samples in the majority and minority classes. The nonsampling method directly uses the original imbalanced dataset as a baseline control to quantify the impact of imbalance. Undersampling randomly reduces samples from the majority class, which is simple and efficient but may lose key information in the majority class, leading to a decline in the model’s generalization ability. The Synthetic Minority Oversampling Technique (SMOTE) algorithm is a classic oversampling method [22]. It generates new samples through linear interpolation within the neighborhood of minority class samples, which is simple and effective. However, its linear assumption may deviate from the true data distribution.

### CTGAN

**Figure 2.**
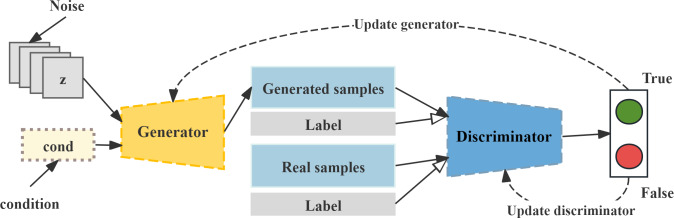
Conditional Tabular Generative Adversarial Network architecture.

CTGAN is a GAN-based method used to model the distribution of tabular data and extract sample rows from this distribution [23]. As shown in Figure 2, CTGAN introduces a mode-specific normalization method to overcome the problems of non-Gaussian and multimodal distributions, and then trains the generator and discriminator in combination with conditional vectors. The generator takes random noise and class labels as input and outputs synthetic samples; the discriminator distinguishes between real and fake samples under the packed GAN framework, in which multiple samples are jointly evaluated to mitigate mode collapse. The training adopts the Wasserstein generative adversarial network with gradient penalty loss, which incorporates a gradient penalty to alleviate mode collapse, ultimately obtaining minority class samples that are highly consistent with the real distribution.

For the CTGAN model, the training configuration was set as follows: epochs=800 and batch_size=250, with a learning rate of 0.0001 applied to both the generator and discriminator. Regarding the network architecture, the generator and discriminator dimensions were both set to (128, 128), the embedding dimension was 64, and the PAC parameter was 5, with GPU acceleration enabled. The training process was completed after 800 epochs, during which the loss curve was recorded for subsequent analysis of training stability.

### TVAE

**Figure 3.**
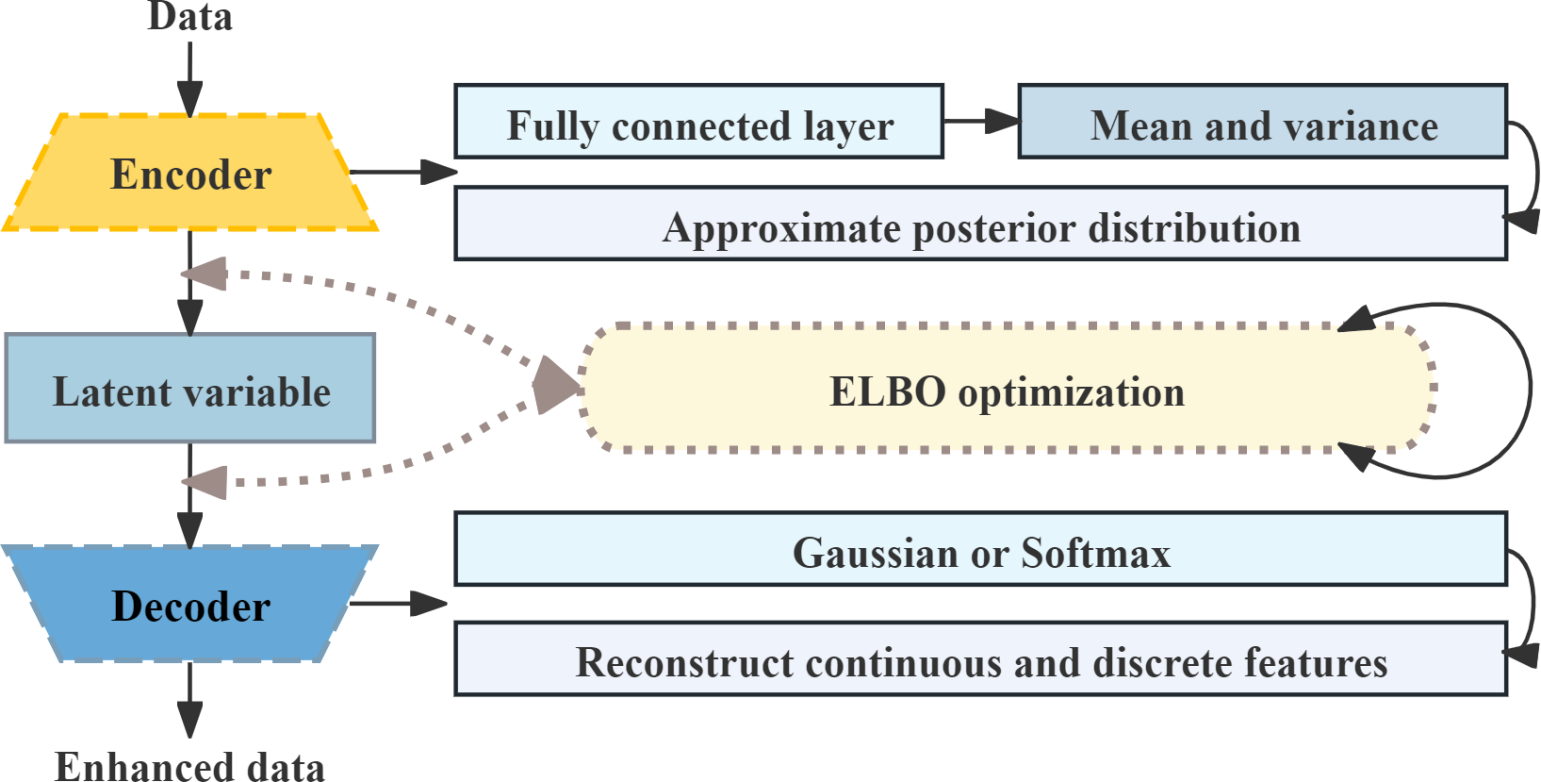
Tabular Variational Autoencoder network structure. ELBO: Evidence Lower Bound.

Figure 3 illustrates the network structure of TVAE. Based on the Variational Autoencoder framework, TVAE captures the complex distribution of tabular data by introducing a probabilistic encoding-decoding structure [24]. The encoder maps raw data to a latent Gaussian distribution, and the decoder reconstructs samples from latent variables while modeling continuous and discrete variables separately. The objective function maximizes the Evidence Lower Bound, balancing reconstruction accuracy and distribution approximation to generate minority class samples that are both realistic and diverse.

The detailed training parameters of TVAE are as follows: epochs=450, batch_size=128, and training was performed on GPU; key model parameters included embedding_dim=256, loss_factor=5, l2scale=0.0001, with compression or decompression dimensions of (512, 256, 128) and (128, 256, 512), respectively. The training was terminated after 450 rounds, and the generator loss was recorded every 10 rounds to monitor training stability.

### LLM-Inspired Feature Enhancement Method

A single laboratory indicator contains limited information, while LLMs can automatically extract unstructured pathological knowledge from literature into structured features, breaking through the reliance of traditional feature engineering on expert experience. In this study, information value is used to measure the predictive contribution of each variable to the outcome, and the Pearson correlation coefficient is used to quantify the linear relationship between continuous variables. Both serve as inputs to the LLM, providing an empirical basis for feature selection.

This study uses a systems biology approach, leveraging the knowledge distillation capabilities of LLMs and statistical methods to generate descriptions of pathological mechanisms and recommend associated metrics. Biomarkers are initially grouped based on related organ systems, followed by expert review, optimization, and adjustment of the groupings, thereby constructing feature aggregations guided by medical knowledge. Some features are divided into three distinct subgroups:

1. Basic Demographic Characteristics Group, encompassing maternal age, gravidity, and parity.
2. Metabolic Syndrome Group (MSG), including FBG, prepregnancy BMI, TG, and total cholesterol.
3. Core Liver Biomarkers Group, comprising γ-glutamyl transferase, cholinesterase, and total bilirubin.

The feature grouping process was operationalized as a human-in-the-loop, LLM-guided conceptual framework. Specifically, LLMs were used as interactive knowledge synthesis engines to mine underlying pathophysiological connections from extensive biomedical literature. Rather than acting as a deterministic algorithmic function, the LLMs generated domain-informed hypotheses for feature aggregation. Ultimately, every proposed feature group was verified through its actual predictive performance within our ML models on the clinical dataset.

Each grouping will calculate corresponding statistics, including mean, SD, maximum value, and range. The mean reflects the overall level of the system, SD assesses the coordination of indicators within the group, the maximum value identifies extreme deviations within the group, and the range quantifies the degree of heterogeneity.

Feature combinations create new higher-order features by integrating multiple original or derived features, revealing complex relationships hidden within the data. This study designed 4 composite features to quantify various metabolic risk factors and their synergistic effects before and during early pregnancy. These features are triglycerides glucose index (TyG), age×prepregnancy BMI (Age_BMI), (FBG+TG)×prepregnancy BMI (FBG+TG_BMI), FBG×prepregnancy BMI (BMI_FBG). The TyG is calculated as follows:


(1) TyG=Ln[TG(mg/dL)×FBG(mg/dL)/2]

### Predictive Model Construction

This study will focus on tree-based models for predicting GDM. We selected 3 ML algorithms, including decision tree, random forest, and Extreme Gradient Boosting (XGBoost), to develop GDM prediction models.

The decision tree recursively splits data based on the principle of minimizing mean squared error. After reaching preset termination conditions, it outputs predictions using the mean value of leaf nodes [25]. The random forest constructs multiple trees through Bootstrap resampling and randomly selects feature subsets at each node for splitting; it improves stability by averaging the outputs of all trees and estimates generalization error using out-of-bag samples [26]. XGBoost incorporates regularization into the gradient boosting framework, iteratively fits residuals from previous rounds, uses a greedy algorithm to find optimal split points, and gradually accumulates outputs from each tree, thereby reducing overfitting risks and improving accuracy [27]. These models were implemented in Python using the Scikit-learn and XGBoost libraries.

The models were fed with specified features, and hyperparameter tuning was performed using the RandomizedSearchCV method. This method randomly samples 20 hyperparameter combinations from a predefined parameter distribution space, with *F*_1_-score as the optimization target. It uses 10-fold cross-validation to ensure the model’s generalization performance and selects the parameter combination with the best evaluation results to train the final model. Unlike GridSearchCV, which attempts all possible parameter combinations, this approach randomly samples a subset of combinations from the parameter space. It significantly reduces search time and computational resource consumption without compromising performance.

### Performance Evaluation Metrics

This study aims to predict the occurrence of GDM using ML models and evaluate the models with multiple assessment metrics. The model performance is evaluated through the following metrics: accuracy, recall, precision (positive predictive value), and *F*_1_-score. We also use the AUROC as one of the evaluation metrics. AUROC is used to assess the model’s performance across different thresholds. The closer the AUROC value is to 1, the better the predictive model is at distinguishing between individuals with and without GDM.

### Interpretable Analysis

The purpose of model interpretability analysis methods is to visualize the reasoning or prediction process of ML models through visual images and other means, helping people intuitively understand why a model makes a certain decision. Especially in the task of predicting GDM, interpreting and verifying the model’s prediction results is conducive to clinical diagnosis and decision-making.

Shapley additive explanations (SHAP) analysis, as a model interpretation method based on the game theory framework, enhances the interpretability of ML models systematically by decomposing the model’s output results and assigning feature importance scores for specific predictions to each input feature [28]. A positive score indicates that the feature plays a supporting role in the model’s prediction results, while a negative score means the feature has an inhibitory effect on the prediction results. With the SHAP method, people can clearly analyze the model’s output mechanism, identify the core features that contribute the most to the prediction results, and quantify the direction and extent of the features’ influence, ultimately achieving a transparent interpretation of the model’s prediction logic [9,29].

### Ethical Considerations

This study was approved by the Medical Ethics Committee of Huizhou First Maternal and Child Health Hospital (ethics approval 20240328A14), and all pregnant women signed informed consent forms.

## Results

### Experimental Setup

Both baseline dataset A and baseline dataset B underwent the same preprocessing procedure. They were used for training CTGAN and TVAE as well as for developing the GDM prediction model. Specifically, baseline dataset A contained 5251 samples, including 966 (18.4%) positive cases, while baseline dataset B contained 2963 samples, including 598 (20.2%) positive cases. To alleviate class imbalance, the study compared 5 strategies: nonsampling, undersampling, SMOTE, CTGAN, and TVAE, with a focus on optimizing the GAN workflow. Specifically, GAN networks were used to model the training dataset and generate new data at a certain proportion; positive samples from the generated new data were merged into the original training dataset for the first data balancing; subsequently, undersampling was applied for the second balancing to ensure complete balance in both quantity and distribution of the dataset.

Both datasets retained 36 original features, including demographic, physiological, and laboratory indicators. Notably, the feature enhancement method was divided into 2 stages: in the first stage, 4 statistical metrics were generated for each of the 3 groups (Basic Demographic Characteristics Group, MSG, and Core Liver Biomarkers Group), resulting in a total of 12 new features; in the second stage, 4 combined features were further constructed to explore nonlinear correlations.

### Clinical Rationality of Synthetic Data

To ensure the reliability of synthetic data, this study conducted rigorous validation from 2 dimensions: statistical distribution and clinical plausibility. First, regarding the validity of individual variable values, by verifying whether synthetic features fall within the clinically acceptable range of real data, the results showed that TVAE achieved clinical compliance rates of 0.979 and 0.988 in the full sample and positive samples, respectively, which were significantly higher than those of CTGAN (0.906 and 0.922). Second, in terms of feature correlation consistency, the data structure was evaluated using the mean absolute error of pairwise correlation coefficients. TVAE exhibited mean absolute error values of 0.0325 and 0.0421 in the full sample and positive samples, both lower than CTGAN values of 0.0622 and 0.0685, indicating that TVAE can more effectively preserve the multivariate statistical structure of the original data.

The physiological plausibility of the synthetic samples was rigorously verified through independent blinded evaluations by clinical domain experts. Senior obstetricians independently evaluated a dataset mixed with real and synthetic samples, scoring the clinical plausibility of each sample (on a 1‐4 scale) and attempting to identify whether samples were real or synthetic. The results indicated that TVAE-generated samples had a higher average plausibility score than CTGAN-generated samples. Moreover, these experts found it difficult to effectively distinguish TVAE synthetic samples from real samples, demonstrating high simulation fidelity. Based on the multidimensional validation results, it is confirmed that synthetic samples generated by TVAE possess both statistical consistency and clinical validity, with data quality superior to that of CTGAN.

### Model Results

To ensure the stability and reliability of the prediction models, the evaluation was conducted in 2 phases, with each phase comprising 3 independent assessments: training using only the original features, training with the addition of organ statistical features, and training with the integration of medical knowledge features. In Phase I, dataset A was used as the training set with 10-fold cross-validation to build the prediction models, while dataset B served as the test set for performance evaluation. In Phase II, the roles of the datasets were reversed, with dataset B as the training set and dataset A as the test set.

**Table 1.** Evaluation results of models using only original features (Phase I).

Models and methods	Train samples	Test samples	Accuracy	Recall	Precision	*F*_1_-score	AUROC^a^
XGBoost^b^							
Baseline^c^	5251	2963	0.8765	0.5184	0.7990	0.6288	0.8555
Undersampled	1932	2963	0.7398	0.8278	0.4256	0.5622	0.8459
SMOTE^d^	8570	2963	0.8994	0.6355	0.8261	0.7183	0.9112
CTGAN^e^	8566	2963	0.8113	0.4281	0.5412	0.4781	0.7810
TVAE^f^	7754	2963	0.8761	0.7140	0.6854	0.6994	0.9015
Random forest							
Baseline	5251	2963	0.8616	0.3261	0.9653	0.4875	0.8826
Undersampled	1932	2963	0.6905	0.7174	0.3645	0.4834	0.7733
SMOTE	8570	2963	0.8653	0.7308	0.6474	0.6866	0.8931
CTGAN	8566	2963	0.8556	0.5953	0.6568	0.6246	0.8517
TVAE	7754	2963	0.8201	0.7157	0.5411	0.6163	0.8559
Decision tree							
Baseline	5251	2963	0.8171	0.3980	0.5667	0.4676	0.7784
Undersampled	1932	2963	0.7172	0.4465	0.3450	0.3892	0.6666
SMOTE	8570	2963	0.7688	0.6271	0.4480	0.5226	0.7716
CTGAN	8566	2963	0.7617	0.3880	0.4056	0.3966	0.6807
TVAE	7754	2963	0.6895	0.5151	0.3284	0.4010	0.6627

a AUROC: area under the receiver operating characteristic curve.

b XGBoost: Extreme Gradient Boosting.

c Baseline: no resampling.

d SMOTE: Synthetic Minority Oversampling Technique.

e CTGAN: Conditional Tabular Generative Adversarial Network.

f TVAE: Tabular Variational Autoencoder.

Tables1^3^ present the evaluation results of models using different features in the first stage. In terms of data augmentation, except for the CTGAN method that only used the original features, models applying augmentation methods all achieved significant improvements in recall compared with the baseline. Models using the undersampling method showed particularly notable gains in recall. In Phase II, the recall of the random forest model reached as high as 0.8344. However, this method tended to show a decline in *F*_1_-score and accuracy compared with the baseline. This conclusion also applies to all evaluations of XGBoost and decision tree models. Models using the TVAE method also achieved remarkable improvements in recall, second only to the undersampling method. In particular, for all evaluations of the XGBoost model, the increase in recall brought by TVAE exceeded that of the SMOTE method. At the same time, TVAE had a weaker negative impact on *F*_1_-score and accuracy than undersampling and could even improve *F*_1_-score in some cases.

**Table 2.** Evaluation results of models introducing organ statistical features (Phase I).

Models and methods	Train samples	Test samples	Accuracy	Recall	Precision	*F*_1_-score	AUROC^a^
XGBoost^b^							
Baseline^c^	5251	2963	0.9015	0.5769	0.8984	0.7026	0.8836
Undersampled	1932	2963	0.7351	0.8211	0.4200	0.5557	0.8371
SMOTE^d^	8570	2963	0.9035	0.6488	0.8362	0.7307	0.9178
CTGAN^e^	8566	2963	0.8201	0.4448	0.5696	0.4995	0.7882
TVAE^f^	7754	2963	0.8147	0.6957	0.5313	0.6025	0.8428
Random forest							
Baseline	5251	2963	0.8630	0.3462	0.9324	0.5049	0.8854
Undersampled	1932	2963	0.7243	0.8344	0.4100	0.5499	0.8491
SMOTE	8570	2963	0.8596	0.7191	0.6342	0.6740	0.8951
CTGAN	8566	2963	0.8569	0.6187	0.6537	0.6357	0.8548
TVAE	7754	2963	0.8458	0.7508	0.5931	0.6627	0.8833
Decision tree							
Baseline	5251	2963	0.8167	0.4448	0.5577	0.4949	0.7605
Undersampled	1932	2963	0.5089	0.7458	0.2550	0.3801	0.6391
SMOTE	8570	2963	0.7685	0.6722	0.4507	0.5396	0.7722
CTGAN	8566	2963	0.7665	0.5234	0.4347	0.4750	0.7601
TVAE	7754	2963	0.4857	0.7876	0.2521	0.3820	0.6276

a AUROC: area under the receiver operating characteristic curve.

b XGBoost: Extreme Gradient Boosting.

c Baseline: no resampling.

d SMOTE: Synthetic Minority Oversampling Technique.

e CTGAN: Conditional Tabular Generative Adversarial Network.

f TVAE: Tabular Variational Autoencoder.

**Table 3.** Evaluation results of models integrating medical knowledge features (Phase I).

Models and methods	Train samples	Test samples	Accuracy	Recall	Precision	*F*_1_-score	AUROC^a^
XGBoost^b^							
Baseline^c^	5251	2963	0.8788	0.5552	0.7812	0.6491	0.8762
Undersampled	1932	2963	0.7324	0.8311	0.4180	0.5562	0.8462
SMOTE^d^	8570	2963	0.9038	0.6522	0.8351	0.7324	0.9136
CTGAN^e^	8566	2963	0.8873	0.7124	0.7245	0.7184	0.9018
TVAE^f^	7754	2963	0.8758	0.7274	0.6797	0.7027	0.9084
Random forest							
Baseline	5251	2963	0.8687	0.3645	0.9604	0.5285	0.8792
Undersampled	1932	2963	0.6841	0.7391	0.3617	0.4857	0.7832
SMOTE	8570	2963	0.8498	0.7358	0.6052	0.6642	0.8923
CTGAN	8566	2963	0.8404	0.6237	0.6006	0.6120	0.8411
TVAE	7754	2963	0.8444	0.7559	0.5893	0.6623	0.8873
Decision tree							
Baseline	5251	2963	0.8137	0.4783	0.5437	0.5089	0.7661
Undersampled	1932	2963	0.6473	0.6003	0.3082	0.4073	0.6780
SMOTE	8570	2963	0.7617	0.6505	0.4391	0.5243	0.7738
CTGAN	8566	2963	0.7648	0.5518	0.4348	0.4864	0.7485
TVAE	7754	2963	0.5886	0.6271	0.2735	0.3809	0.6449

a AUROC: area under the receiver operating characteristic curve.

b XGBoost: Extreme Gradient Boosting.

c Baseline: no resampling.

d SMOTE: Synthetic Minority Oversampling Technique.

e CTGAN: Conditional Tabular Generative Adversarial Network.

f TVAE: Tabular Variational Autoencoder.

Tables 4-6 present the evaluation results of models using different features in the second phase, aimed at verifying the models’ generalization capability. It can be observed that the conclusions drawn from Table 1 also apply here. Notably, when comparing the evaluation results of Phase I and Phase II, we found that for the random forest model using the TVAE method, the recall rate in Phase I increased progressively from 0.7157 in assessment I to 0.7508 in assessment II and then to 0.7559 in assessment III. The same trend in recall rate improvement was also observed for the model in Phase II after the introduction of new features.

**Table 4.** Evaluation results of models using only original features (Phase II).

Models and methods	Train samples	Test samples	Accuracy	Recall	Precision	*F*_1_-score	AUROC^a^
XGBoost^b^							
Baseline^c^	2963	5251	0.8608	0.4576	0.6810	0.5474	0.7893
Undersampled	1196	5251	0.7149	0.7246	0.3625	0.4833	0.7850
SMOTE^d^	4730	5251	0.8463	0.5321	0.5915	0.5602	0.8209
CTGAN^e^	8666	5251	0.8469	0.5342	0.5931	0.5621	0.8135
TVAE^f^	6328	5251	0.8324	0.5994	0.5401	0.5682	0.8384
Random forest							
Baseline	2963	5251	0.8581	0.2567	0.9018	0.3997	0.8203
Undersampled	1196	5251	0.7020	0.7091	0.3479	0.4668	0.7744
SMOTE	4730	5251	0.8096	0.6077	0.4859	0.5400	0.8156
CTGAN	8666	5251	0.8094	0.5507	0.4841	0.5153	0.7746
TVAE	6328	5251	0.7793	0.6812	0.4361	0.5317	0.8193
Decision tree							
Baseline	2963	5251	0.7976	0.3602	0.4388	0.3957	0.7097
Undersampled	1196	5251	0.6109	0.6035	0.2599	0.3634	0.6590
SMOTE	4730	5251	0.6203	0.6739	0.2794	0.3950	0.6828
CTGAN	8666	5251	0.7001	0.5166	0.3105	0.3879	0.6784
TVAE	6328	5251	0.6376	0.6108	0.2787	0.3827	0.6745

a AUROC: area under the receiver operating characteristic curve.

b XGBoost: Extreme Gradient Boosting.

c Baseline: no resampling.

d SMOTE: Synthetic Minority Oversampling Technique.

e CTGAN: Conditional Tabular Generative Adversarial Network.

f TVAE: Tabular Variational Autoencoder.

**Table 5.** Evaluation results of models introducing organ statistical features (Phase II).

Models and methods	Train samples	Test samples	Accuracy	Recall	Precision	*F*_1_-score	AUROC^a^
XGBoost^b^							
Baseline^c^	2963	5251	0.8541	0.2277	0.9167	0.3648	0.8185
Undersampled	1196	5251	0.7119	0.7246	0.3595	0.4806	0.7907
SMOTE^d^	4730	5251	0.8459	0.5362	0.5893	0.5615	0.8210
CTGAN^e^	8666	5251	0.8259	0.5145	0.5276	0.5210	0.7869
TVAE^f^	6328	5251	0.7616	0.6656	0.4090	0.5067	0.8023
Random forest							
Baseline	2963	5251	0.8530	0.2277	0.8943	0.3630	0.8270
Undersampled	1196	5251	0.7052	0.7308	0.3541	0.4770	0.7903
SMOTE	4730	5251	0.8065	0.6242	0.4801	0.5428	0.8101
CTGAN	8666	5251	0.8077	0.5466	0.4800	0.5111	0.7836
TVAE	6328	5251	0.7701	0.7039	0.4247	0.5298	0.8195
Decision tree							
Baseline	2963	5251	0.7924	0.3406	0.4207	0.3764	0.6880
Undersampled	1196	5251	0.6757	0.4565	0.2724	0.3412	0.6390
SMOTE	4730	5251	0.6165	0.6646	0.2753	0.3893	0.6627
CTGAN	8666	5251	0.7220	0.5228	0.3358	0.4089	0.6937
TVAE	6328	5251	0.6905	0.6460	0.3272	0.4344	0.7181

a AUROC: area under the receiver operating characteristic curve.

b XGBoost: Extreme Gradient Boosting.

c Baseline: no resampling.

d SMOTE: Synthetic Minority Oversampling Technique.

e CTGAN: Conditional Tabular Generative Adversarial Network.

f TVAE: Tabular Variational Autoencoder.

**Table 6.** Evaluation results of models integrating medical knowledge features (Phase II).

Models and methods	Train samples	Test samples	Accuracy	Recall	Precision	*F*_1_-score	AUROC^a^
XGBoost^b^							
Baseline^c^	2963	5251	0.8659	0.4172	0.7408	0.5338	0.7926
Undersampled	1196	5251	0.7041	0.7236	0.3520	0.4736	0.7908
SMOTE^d^	4730	5251	0.8448	0.5507	0.5827	0.5663	0.8246
CTGAN^e^	8666	5251	0.8448	0.5466	0.5834	0.5644	0.8162
TVAE^f^	6328	5251	0.8284	0.6315	0.5281	0.5752	0.8371
Random forest							
Baseline	2963	5251	0.8619	0.2992	0.8576	0.4436	0.8199
Undersampled	1196	5251	0.6999	0.6988	0.3444	0.4614	0.7724
SMOTE	4730	5251	0.7987	0.6325	0.4653	0.5362	0.8114
CTGAN	8666	5251	0.8056	0.5642	0.4760	0.5163	0.7798
TVAE	6328	5251	0.7734	0.7143	0.4302	0.5370	0.8234
Decision tree							
Baseline	2963	5251	0.7993	0.3675	0.4449	0.4025	0.7101
Undersampled	1196	5251	0.5309	0.7464	0.2453	0.3693	0.6314
SMOTE	4730	5251	0.7250	0.5559	0.3460	0.4265	0.7058
CTGAN	8666	5251	0.7313	0.5580	0.3539	0.4331	0.6984
TVAE	6328	5251	0.6254	0.6532	0.2788	0.3908	0.6869

a AUROC: area under the receiver operating characteristic curve.

b XGBoost: Extreme Gradient Boosting.

c Baseline: no resampling.

d SMOTE: Synthetic Minority Oversampling Technique.

e CTGAN: Conditional Tabular Generative Adversarial Network.

f TVAE: Tabular Variational Autoencoder.

To validate the effectiveness of the proposed framework combining GAN-based data augmentation with LLM-inspired feature enhancement, this study systematically evaluated the impact of different strategies on model performance using statistical methods. First, a nonparametric Kruskal-Wallis test was conducted to determine whether data augmentation methods and feature enhancement methods produced statistically significant differences in model performance metrics such as accuracy and recall [30]. When the Kruskal-Wallis test indicated significant differences, pairwise comparisons were further performed using the Games-Howell post hoc test to identify which specific augmentation strategies led to superior performance. Meanwhile, Cohen *d* effect size was used to quantify the practical significance of the differences: an effect size of 0.2‐0.5 indicated a small difference, 0.5‐0.8 a medium difference, and greater than 0.8 a large difference [31], thereby distinguishing whether statistically significant differences also had practical value.

Table 7 presents the results of the Kruskal-Wallis test. In this test, H denotes the Kruskal-Wallis test statistic, which is used to evaluate whether significant differences exist among multiple independent groups. For data augmentation methods, significant effects were observed on accuracy (*P*<.001), recall (*P*<.001), precision (*P*<.001), and *F*_1_-score (*P*=.04), with corresponding effect sizes of 0.406, 0.563, 0.457, and 0.147, respectively. Specifically, the effect sizes of accuracy, recall, and precision indicated a strong impact of data augmentation methods, while the effect size of *F*_1_-score suggested a moderate impact. No significant effect of data augmentation methods was observed on AUROC (Kruskal-Wallis H=8.099, *P*=.09) with a small effect size ($\eta^{2}$=0.102). In contrast, feature enhancement methods did not have a significant impact on any of the evaluated metrics, and their effect sizes were negligible ($\eta^{2}$≤0.010).

**Table 7.** Kruskal-Wallis test results.

Metric and factor	Statistic	Effect size (*η*^2^)	*P* value
Accuracy			
Feature enhancement	0.055	0.000	.97
Data augmentation	20.257	0.406	≤.001
Recall			
Feature enhancement	2.429	0.010	.30
Data augmentation	26.510	0.563	<.001
Precision			
Feature enhancement	0.033	0.000	.98
Data augmentation	22.285	0.457	≤.001
*F*_1_-score			
Feature enhancement	0.842	0.000	.66
Data augmentation	9.864	0.147	.04
AUROC^a^			
Feature enhancement	0.281	0.000	.87
Data augmentation	8.099	0.102	.09

a AUROC: area under the receiver operating characteristic curve.

**Figure 4.**
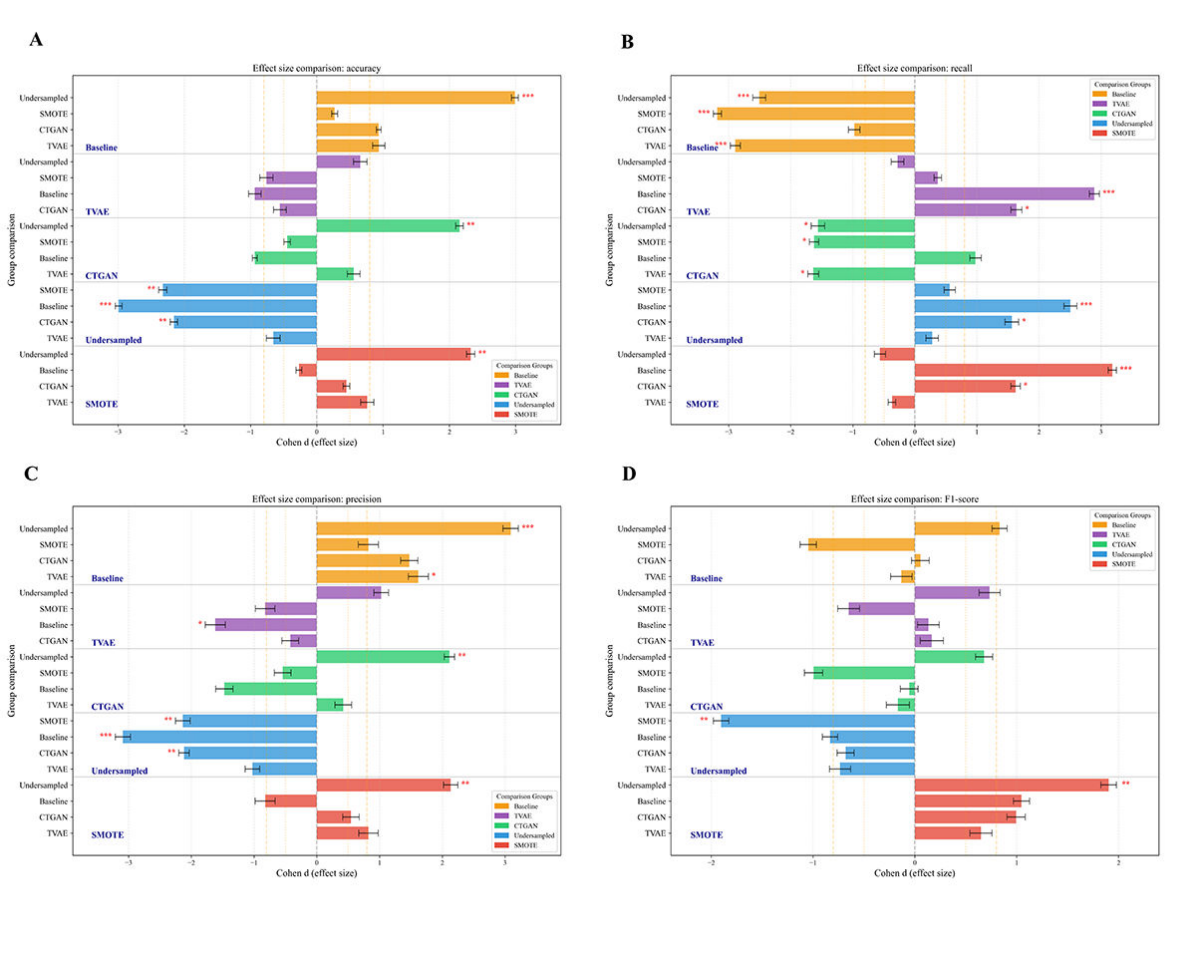
Forest plot comparing the predictive performance: (A) accuracy, (B) recall, (C) precision, (D) *F*_1_-score (asterisks denote statistical significance levels where * represents *P*<.05, ** represents *P*<.01, and *** represents *P*<.001). Baseline: no resampling; CTGAN: Conditional Tabular Generative Adversarial Network; SMOTE: Synthetic Minority Oversampling Technique; TVAE: Tabular Variational Autoencoder.

For the metrics that showed significance in the Kruskal-Wallis test, group differences were further analyzed using the Games-Howell post hoc test. Figure 4 presents the results as a forest plot. Recall is a core metric for GDM prediction, reflecting the model’s ability to identify positive cases and avoid missed diagnoses. The recall of TVAE was significantly higher than that of the baseline (0.4454, *P*<.001) and CTGAN (0.5429, *P*=.02), with large effect sizes (Cohen *d*=2.894 and 1.637, respectively), indicating that TVAE-generated samples effectively enhance the model’s recognition of positive cases. For accuracy, SMOTE (0.8423) and TVAE (0.7601) did not differ significantly (*P*=.52), but both were significantly higher than undersampling (0.6866, *P*<.01), indicating that generated samples did not compromise overall classification ability.

Traditional oversampling methods, such as SMOTE, demonstrate a clear advantage in rapidly increasing recall. However, their critical weakness lies in generating boundary noise via linear interpolation within the highly nonlinear metabolic feature space, often leading to a disproportionate drop in precision. While CTGAN mitigates this issue, it exhibits limitations when processing continuous, heavily skewed clinical biomarkers. In contrast, the TVAE approach demonstrates a distinct superiority. By modeling a continuous latent Gaussian space, it smoothly reconstructs physiological variables, achieving a superior balance between sensitivity and specificity. Furthermore, when original features are augmented with LLM-inspired composite features, the framework exhibits a remarkable capability in capturing synergistic pathological interactions, which nonlinear tree–based models leverage to build highly robust decision boundaries. The inherent weakness of this dual-enhanced, high-recall strategy is a marginal increase in the false-positive rate. Nevertheless, in the clinical context of early first-trimester screening, the benefit of capturing nearly all true high-risk cases for noninvasive lifestyle intervention significantly outweighs the easily manageable drawback of transient false-positive alerts.

In summary, we consider that the random forest model enhanced by TFRFM represents an effective approach for early-pregnancy prediction of GDM, providing strong support for clinical decision-making. This model demonstrated stable and superior performance across different data augmentation and feature enhancement strategies, showing high accuracy and AUROC. Notably, it effectively improved the identification of positive cases while maintaining a balance between recall and precision, which is critical for the early prediction and precise management of GDM.

### Interpretability Analysis Results: Feature Importance and Correlation Analysis

To investigate the role of features in the model, we conducted feature importance and correlation analyses using the TFRFM. By calculating each feature’s contribution to the model’s decision-making process, we identified the top 20 features that play a critical role in predicting early-pregnancy GDM. Additionally, correlation analysis was performed on these 20 features to reveal potential relationships and interactions among them.

**Figure 5.**
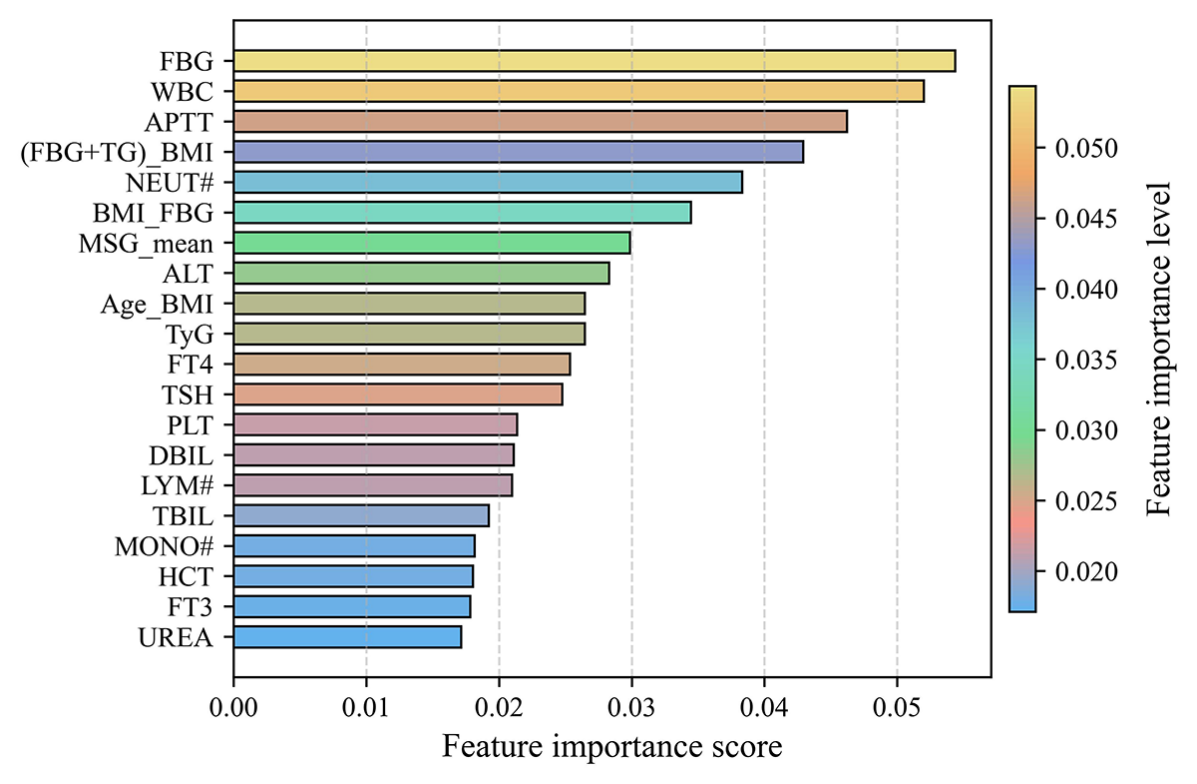
Feature importance analysis of the Tabular Variational Autoencoder–based feature augmentation. Age_BMI: age×prepregnancy BMI; ALT: alanine aminotransferase; APTT: activated partial thromboplastin time; BMI_FBG: fasting blood glucose×prepregnancy BMI; DBIL: direct bilirubin; FBG: fasting blood glucose; (FBG+TG)_BMI: (fasting blood glucose+triglycerides)×prepregnancy BMI; FT3: free triiodothyronine; FT4: free thyroxine; HCT: hematocrit; LYM#: lymphocytes; MONO#: monocytes; MSG_mean: the mean of the metabolic syndrome group; NEUT#: neutrophil; PLT: platelet count; TBIL: total bilirubin; TSH: thyroid-stimulating hormone; TyG: triglycerides glucose index; WBC: white blood cell count.

Figure 5 presents the feature importance analysis of the TFRFM. FBG achieved the highest importance score, followed by white blood cell count (WBC) and activated partial thromboplastin time (APTT), indicating that these features contribute substantially to the model’s decision-making process and can be regarded as the most critical predictors of GDM. In addition, the 4 composite features investigated in this study, along with the mean of MSG, were also ranked among the top 20 features, demonstrating their important role in prediction and further validating the effectiveness of integrating medically informed features. Notably, although some traditional indicators such as age, TG, and BMI may not appear prominent when analyzed independently, their contributions become substantial when combined with other features or when potential interfeature correlations are considered. This finding highlights that interactions and relationships among features are also crucial for accurately predicting early-pregnancy GDM.

**Figure 6.**
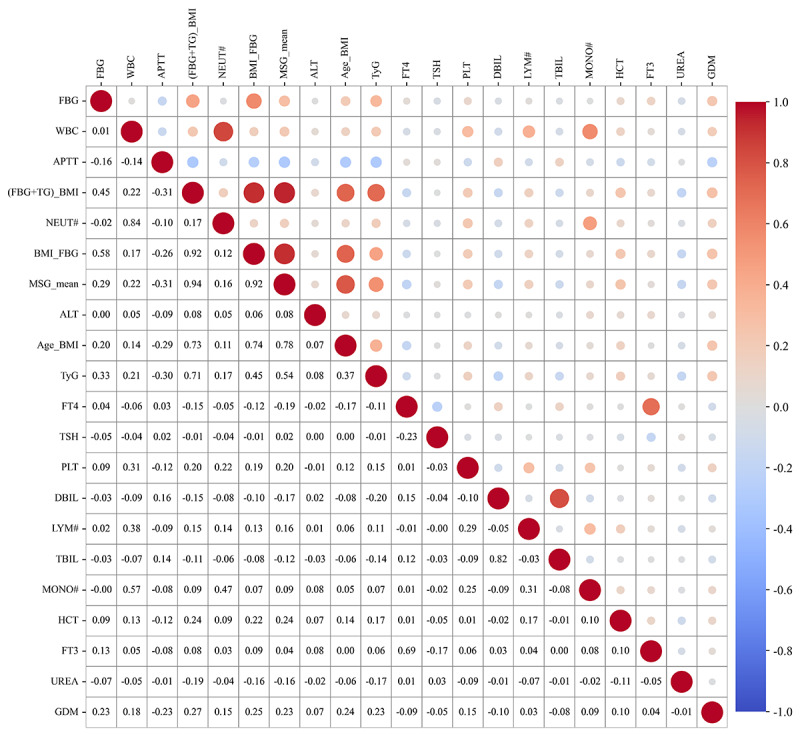
Correlation analysis of the top 20 important features in the Tabular Variational Autoencoder–based feature augmentation. Age_BMI: age×prepregnancy BMI; ALT: alanine aminotransferase; APTT: activated partial thromboplastin time; BMI_FBG: fasting blood glucose×prepregnancy BMI; DBIL: direct bilirubin; FBG: fasting blood glucose; (FBG+TG)_BMI: (fasting blood glucose+triglycerides)×prepregnancy BMI; FT3: free triiodothyronine; FT4: free thyroxine; HCT: hematocrit; LYM#: lymphocytes; MONO#: monocytes; MSG_mean: the mean of the metabolic syndrome group; NEUT#: neutrophil; PLT: platelet count; TBIL: total bilirubin; TSH: thyroid-stimulating hormone; TyG: triglycerides glucose index; WBC: white blood cell count.

Figure 6 also presents the correlation analysis among these 20 features. The size of the circles represents the absolute magnitude of the correlation coefficients, and the color gradient corresponds to the direction and strength of the correlations, with red indicating a positive correlation and blue indicating a negative correlation, where darker shades represent stronger associations. The composite feature (FBG+TG)_BMI, ranked fourth in feature importance, exhibited the highest Pearson correlation with GDM, followed by BMI_FBG, Age_BMI, TyG, MSG_mean, FBG, APTT, and WBC, with APTT showing a negative correlation with GDM. These correlation results suggest that the relationships among combined features and their association with GDM may be more complex and informative than analyses of individual features alone. This underscores the importance of considering interfeature interactions and associations when predicting early-pregnancy GDM rather than relying solely on the significance of individual features.

### SHAP Analysis Results

SHAP analysis quantifies the contribution of each feature to the model’s predictions, providing an intuitive view of feature importance rankings and influence patterns under different methods. In this section, we focus on comparing the SHAP analysis results of the TFRFM, the random forest model enhanced by the TVAE method (TVRFM), and the random forest model trained on baseline data (BLRFM), highlighting the advantages of integrating the TVAE approach with feature enhancement in capturing key predictive factors, stabilizing feature effects, and characterizing feature relationships.

**Figure 7.**
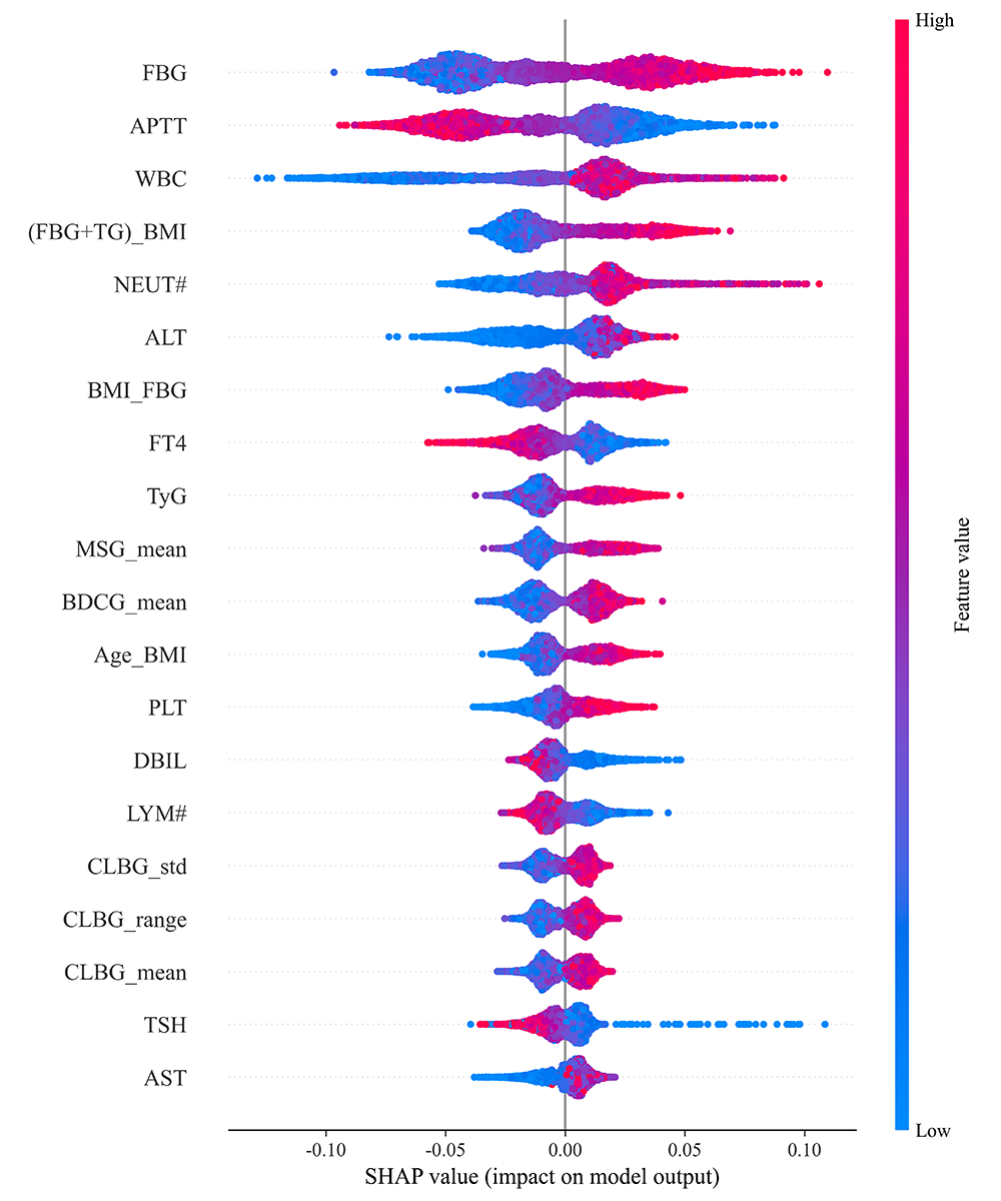
Shapley additive explanations beeswarm plot of the Tabular Variational Autoencoder–based feature augmentation. Age_BMI: age×prepregnancy BMI; ALT: alanine aminotransferase; APTT: activated partial thromboplastin time; AST: aspartate aminotransferase; BDCG_mean: the mean of the basic demographic characteristics group; BMI_FBG: fasting blood glucose×prepregnancy BMI; CLBG_mean: the mean of the core liver biomarkers group; CLBG_range: the range of the core liver biomarkers group; CLBG_std: the standard deviation of the core liver biomarkers group; DBIL: direct bilirubin; FBG: fasting blood glucose; (FBG+TG)_BMI: (fasting blood glucose+triglycerides)×prepregnancy BMI; FT4: free thyroxine; LYM#: lymphocytes; MSG_mean: the mean of the metabolic syndrome group; NEUT#: neutrophil; PLT: platelet count; SHAP: Shapley additive explanations; TSH: thyroid-stimulating hormone; TyG: triglycerides glucose index; WBC: white blood cell count.

As shown in Figure 7, the top 5 features in terms of average SHAP values for the TFRFM are FBG, APTT, WBC, (FBG+TG)_BMI, and neutrophil (NEUT#), indicating that these are the key drivers for predicting blood glucose levels in the model. In terms of the magnitude of feature impact, FBG has a SHAP range of (–0.10 to 0.125) with a span of 0.225, and WBC has a SHAP range of (−0.125 to 0.10) with the same span of 0.225, showing the greatest fluctuations in prediction influence. This aligns with clinical understanding, where blood glucose (eg, FBG) and inflammatory indicators (eg, WBC) are directly associated with pregnancy risk. Figure 7 clearly shows that the average SHAP values of the top 20 features are all greater than 0, indicating that these features contribute positively to the prediction, and the feature importance ranking aligns well with medical knowledge.

**Figure 8.**
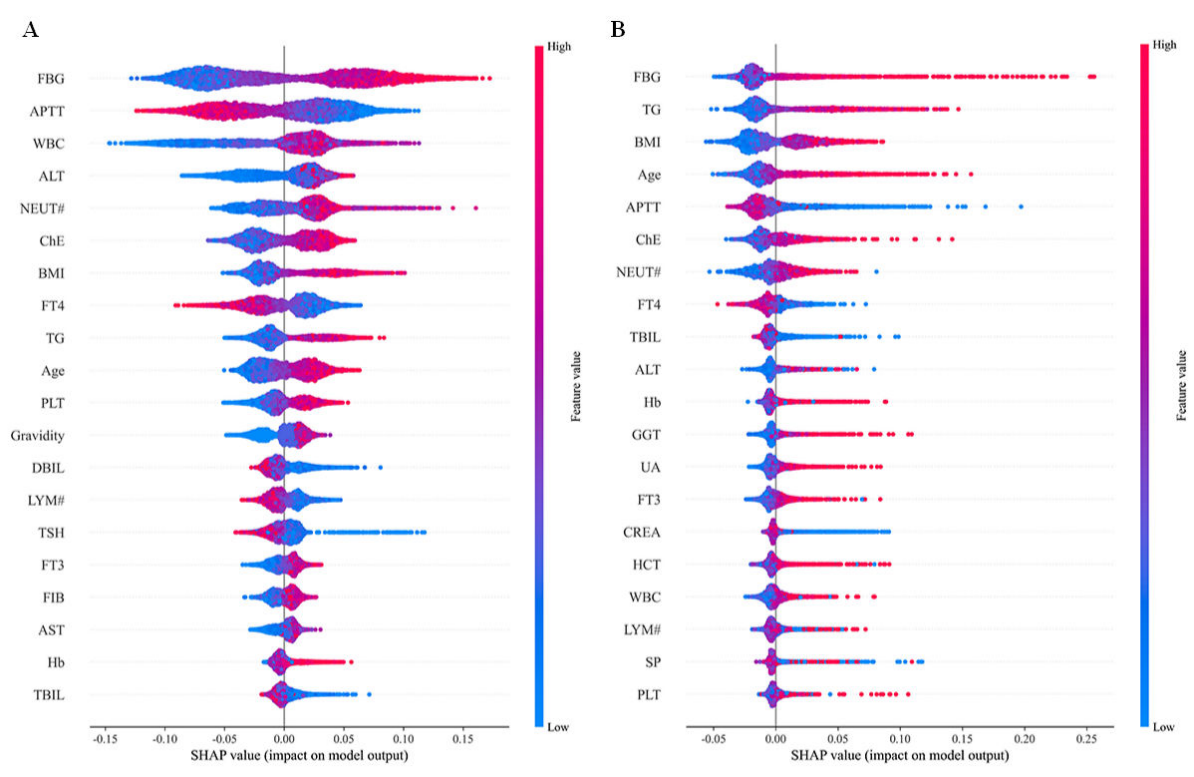
Shapley additive explanations beeswarm plots of Tabular Variational Autoencoder–based feature augmentation (**A**) and random forest model trained on baseline data (**B**). ALT: alanine aminotransferase; APTT: activated partial thromboplastin time; AST: aspartate aminotransferase; ChE: cholinesterases; CREA: creatinine; DBIL: direct bilirubin; FBG: fasting blood glucose; FIB: fibrinogen; FT3: free triiodothyronine; FT4: free thyroxine; GCT: γ-glutamyl transferase; Hb: hemoglobin; HCT: hematocrit; LYM#: lymphocytes; NEUT#: neutrophil; PLT: platelet count; SHAP: Shapley additive explanations; SP: systolic pressure; TBIL: total bilirubin; TG: triglycerides; TSH: thyroid-stimulating hormone; TVAE: Tabular Variational Autoencoder; UA: uric acid; WBC: white blood cell count.

Figure 8 shows the SHAP beeswarm plots for TVRFM and BLRFM. The top 5 features by average SHAP value in TVRFM are FBG, APTT, WBC, ALT, and NEUT#, with an overlap of only 9 out of 20 (45%) with the top 20 features in TFRFM. In TVRFM, the SHAP value range for FBG is 0.3 (−0.125 to 0.175) and for WBC is 0.275 (−0.15 to 0.125), both larger than in TFRFM, indicating greater variability of key feature influence and lower model stability without feature enhancement. In BLRFM, the top 5 features by average SHAP value are FBG, TG, BMI, age, and APTT, with an overlap of only 7 out of 20 (35%) with the top 20 features in TFRFM. Notably, inflammation-related indicators such as WBC and NEUT# are absent, while age, which has weaker direct clinical relevance, is included. In the baseline model, the SHAP value range for FBG reaches 0.325 (−0.05 to 0.275), exhibiting the largest fluctuation. The scatter points are elongated on the right side of the zero line and concentrated on the left, indicating substantial prediction bias for high-risk pregnant women.

The above findings indicate that the introduction of enhanced features such as (FBG+TG)_BMI strengthens the impact of interactions among physiological indicators, aligning more closely with clinical logic. The SHAP value fluctuations of key features, including FBG and WBC, are reduced, improving the robustness of model predictions. The model also decreases the presence of irregular feature-target relationships, captures linear associations more clearly, and retains the ability to indicate potential nonlinear relationships. In summary, TFRFM, through the combination of feature enhancement and generative modeling, significantly improves the identification of key predictive factors and enhances model stability, providing a more reliable reference for assessing pregnancy-related risks.

## Discussion

### Principal Findings

The primary finding of this study is that integrating GAN-based data augmentation with LLM-inspired feature enhancement successfully addresses the persistent challenges of class imbalance and complex feature interactions in early-pregnancy GDM prediction. Furthermore, our interpretability analysis revealed that early GDM risk is most strongly driven not only by independent glycemic markers, but by complex metabolic interactions captured through novel composite features, alongside specific inflammatory and coagulation indicators.

Early screening and risk-stratified management of GDM are crucial for reducing the risk of pregnancy-related complications. Effective prediction of GDM in early pregnancy allows for the identification of high-risk pregnant women, enabling clinicians to promptly implement evidence-based interventions. This approach also facilitates the development of precise and individualized management plans tailored to high-risk groups, thereby improving maternal and neonatal health outcomes. Given the profound impact of GDM-related complications on maternal and fetal health, early screening combined with risk-stratified management has become a core component of optimizing GDM interventions [32]. Consequently, exploring earlier and more accurate methods for predicting GDM to enable timely intervention has emerged as an urgent and critical challenge.

The pathogenesis of GDM involves pancreatic β-cell dysfunction and tissue insulin resistance. The combined indicator of FBG, TG, and BMI, namely the (FBG+TG)_BMI proposed in this study, exhibits a close biological association with insulin resistance. Its underlying mechanism primarily reflects the interplay between dysregulated glucose-lipid metabolism and obesity-related pathophysiological processes. FBG serves as a direct marker of insulin sensitivity, and its elevation indicates increased hepatic glucose output due to hepatic insulin resistance as well as impaired peripheral glucose use. Available studies suggest a biologically plausible link between hyperglycemia and dyslipidemia [33]. Hyperglycemia can trigger reactive oxygen species production in pancreatic β-cells, leading to oxidative stress and cellular dysfunction, thereby exacerbating insulin resistance. On this pathological basis, elevated TG levels directly impair glucose metabolism in muscle tissue and inhibit insulin signaling pathways, further aggravating insulin resistance. Additionally, free fatty acids generated from TG hydrolysis can exert lipotoxic effects, further damaging β-cell function. Consequently, FBG and TG jointly drive the progression of metabolic imbalance, forming a vicious cycle of overlapping glucotoxicity and lipotoxicity. BMI, as a core indicator of obesity, amplifies insulin resistance through ectopic fat deposition, proinflammatory factor release, and endoplasmic reticulum stress. The combined effect of FBG, TG, and BMI comprehensively reflects the metabolic burden by superimposing glucotoxicity, lipotoxicity, and obesity-related states. This synergistic metric captures the complex pathophysiology of insulin resistance more accurately than any single indicator. Accordingly, the composite index (FBG+TG)_BMI may serve as a simple surrogate tool for assessing insulin resistance, particularly suitable for early-pregnancy screening to rapidly identify high-risk individuals, thereby providing a biological basis for risk-stratified management and early intervention.

Clinical studies have shown that pregnant women with GDM often exhibit shortened APTT from early to midpregnancy, which is consistent with a mild hypercoagulable state. For example, in GDM pregnancies, prothrombin time and APTT in early pregnancy are significantly reduced, and multivariate analyses indicate that a shorter APTT is an independent predictor of GDM [34]. Studies examining laboratory data from women at 24‐28 weeks of gestation also report that the GDM group shows markedly shorter prothrombin time and APTT and higher fibrinogen levels compared with controls [35]. Research on type 2 diabetes has similarly found that overt diabetes is associated with shortened APTT and elevated fibrinogen, suggesting a general thrombotic tendency in diabetes [36].

GDM is characterized by key pathophysiological features, including insulin resistance, pancreatic β-cell dysfunction, and low-grade inflammation, all of which contribute to a procoagulant state. Under diabetic conditions, hyperglycemia and insulin resistance increase the expression of coagulation factors in the intrinsic pathway, such as FXII, FXI, and FVIII, resulting in APTT shortening [37]. In GDM, FXII levels are significantly elevated, and its critical role in activating the intrinsic coagulation pathway helps explain the observed APTT reduction [38].

WBC and NEUT#, as markers of inflammation, are involved in the development of GDM by mediating chronic low-grade inflammatory responses. Pregnant women with GDM often exhibit insulin resistance alongside dysregulated glucose and lipid metabolism. Elevated blood glucose and free fatty acids activate inflammatory signaling pathways. This activation stimulates immune cells, such as neutrophils, to release proinflammatory factors, thereby inducing a systemic low-grade inflammatory state. Neutrophils, being the most abundant leukocyte subset in peripheral blood, can further exacerbate inhibition of insulin signaling and β-cell dysfunction when activated or functionally impaired. Mild elevations of WBC and NEUT# are observed in early pregnancy among patients with GDM, and their levels correlate with the degree of insulin resistance, poor glycemic control, and adverse pregnancy outcomes. These findings suggest that inflammatory markers may not only be concomitant phenomena in GDM pathophysiology but also actively contribute to disease development by amplifying the inflammatory cascade, providing potential biological evidence for early identification of high-risk populations and the development of anti-inflammatory targeted interventions.

The risk probability of developing GDM in pregnant women can be quantitatively estimated using ML models [39]. Given the notable advantage of ML in handling complex, high-dimensional data, several ML-based predictive models have demonstrated superior performance compared with traditional regression models [40]. For instance, Ye and colleagues [41] constructed GDM prediction models using more than 30 ML algorithms, including decision trees and random forests, and compared them with logistic regression models. Their results confirmed the stronger predictive capability of ML approaches and identified TG, FBG, BMI, and hemoglobin A_1c_ as among the most important predictors. Similarly, Belsti et al [15] developed new models based on the internationally validated Monash GDM model using XGBoost and CatBoost classifiers, achieving optimal discrimination performance with AUROC of 0.92 and 0.93, respectively, while decision curve analysis confirmed the highest clinical utility of these models [15]. Furthermore, our findings indicate that the TFRFM model, enhanced through data augmentation and feature augmentation, maximizes early prediction of GDM, demonstrating superior robustness and generalization ability, with an AUROC of 0.8873 and a recall of 0.7559.

Recall is an indicator that measures a model’s ability to correctly identify positive cases. An improvement in recall means that the model reduces the risk of missed diagnoses, allowing more pregnant women who truly have GDM to be identified in a timely manner, although this may be accompanied by an increase in false positives. Reducing missed diagnoses directly benefits maternal and neonatal health outcomes, as early prevention or diagnosis of GDM enables pregnant women to receive targeted interventions such as personalized nutritional guidance or blood glucose monitoring, thereby lowering the risks of macrosomia and preterm birth. At the same time, although the false-positive rate may increase, nonpharmacological interventions such as dietary management and exercise guidance following a misdiagnosis still provide positive health effects. Their potential adverse impacts can be effectively mitigated through standardized OGTT reassessment in midpregnancy. Nonpharmacological interventions, being the foundation of GDM management, can help pregnant women develop healthy lifestyles and reduce metabolic burden even in cases of misdiagnosis [42]. Furthermore, OGTT reassessment in early pregnancy serves as the final diagnostic reference, ensuring that high-risk individuals receive appropriate scientific interventions while minimizing unnecessary medical treatment and reducing the potential impact of misdiagnosis on maternal and neonatal health. Therefore, an increase in recall promotes the transition of GDM stratified management from a reactive to a proactive approach and allows primary health care resources to be optimally allocated to high-risk populations.

It must be acknowledged that the screening and management of GDM largely depend on the available medical resources and the clinical judgment of health care providers. However, primary hospitals often exhibit considerable variability in these aspects, which limits their capacity to prevent and manage GDM and reduces their ability to provide timely and reliable personalized interventions for pregnant women. Furthermore, given that GDM may share underlying pathophysiological mechanisms with other pregnancy-related disorders, exploring the relationships among these conditions and applying the proposed framework combining GAN-based data augmentation with LLM-inspired feature enhancement to develop integrated prediction and management tools for multiple gestational diseases holds promise for further improving maternal and neonatal health outcomes. Additionally, future research should incorporate multicenter and multiethnic cohorts to further rigorously validate the model’s generalizability across diverse geographical populations.

### Conclusion

This study proposes a framework that combines GAN-based data augmentation with LLM-inspired feature enhancement, effectively addressing data imbalance in GDM datasets and significantly improving model recall. In our experiments, we observed that a random forest model incorporating TVAE and feature enhancement not only increased recall but also maintained high precision, accuracy, and AUROC. The model demonstrated superior overall performance and stability across multiple independent evaluations, highlighting its potential as an effective tool for early-pregnancy GDM prediction. Furthermore, interpretability analysis revealed that FBG, (FBG+TG)_BMI, APTT, WBC, and NEUT# contributed most significantly to GDM prediction. These findings provide important biological evidence to support rapid screening, stratified management, and early intervention for GDM.

The proposed method not only enhances predictive accuracy but also offers new insights into the pathophysiological mechanisms of GDM. By emphasizing interactions among physiological indicators, the model better captures complex relationships within clinical data, which has important implications for guiding clinical practice. In the future, we aim to further optimize this framework and explore its applicability to predicting other pregnancy-related diseases, advancing the prediction and management of gestational disorders and contributing to improved maternal and neonatal health outcomes.

## Abbreviations

(FBG+TG)_BMI: (fasting blood glucose + triglycerides) × prepregnancy BMI
Age_BMI: age×prepregnancy BMI
APTT: activated partial thromboplastin time
AUROC: area under the receiver operating characteristic curve
BLRFM: random forest model trained on baseline data
BMI_FBG: fasting blood glucose×prepregnancy BMI
CTGAN: Conditional Tabular Generative Adversarial Network
FBG: fasting blood glucose
GAN: Generative Adversarial Network
GDM: gestational diabetes mellitus
IADPSG: International Association of Diabetes and Pregnancy Study Groups
LLM: large language model
ML: machine learning
MSG: Metabolic Syndrome Group
OGTT: Oral Glucose Tolerance Test
PeRSonal: Prediction for Risk-Stratified Care for Women with GDM
SHAP: Shapley additive explanations
SMOTE: Synthetic Minority Oversampling Technique
TFRFM: Tabular Variational Autoencoder–based feature augmentation
TG: triglycerides
TVAE: Tabular Variational Autoencoder
TVRFM: random forest model enhanced by the TVAE method
TyG: triglycerides glucose index
WBC: white blood cell count
XGBoost: Extreme Gradient Boosting
